# Mechanisms Driving Macrophage Diversity and Specialization in Distinct Tumor Microenvironments and Parallelisms with Other Tissues

**DOI:** 10.3389/fimmu.2014.00127

**Published:** 2014-03-26

**Authors:** Eva Van Overmeire, Damya Laoui, Jiri Keirsse, Jo A. Van Ginderachter, Adelaida Sarukhan

**Affiliations:** ^1^Myeloid Cell Immunology Laboratory, VIB, Brussels, Belgium; ^2^Lab of Cellular and Molecular Immunology, Vrije Universiteit Brussel, Brussels, Belgium; ^3^Institut national de la santé et de la recherche médicale, Paris, France

**Keywords:** tumor-associated macrophage, TAM heterogeneity, macrophage ontogeny, macrophage proliferation, hypoxia, obesity, atherosclerosis, feto-maternal interface

## Abstract

Macrophages are extremely versatile cells that adopt a distinct phenotype in response to a changing microenvironment. Consequently, macrophages are involved in diverse functions, ranging from organogenesis and tissue homeostasis to recognition and destruction of invading pathogens. In cancer, tumor-associated macrophages (TAM) often contribute to tumor progression by increasing cancer cell migration and invasiveness, stimulating angiogenesis, and suppressing anti-tumor immunity. Accumulating evidence suggests that these different functions could be exerted by specialized TAM subpopulations. Here, we discuss the potential underlying mechanisms regulating TAM specialization and elaborate on TAM heterogeneity in terms of their ontogeny, activation state, and intra-tumoral localization. In addition, parallels are drawn between TAM and macrophages in other tissues. Together, a better understanding of TAM diversity could provide a rationale for novel strategies aimed at targeting the most potent tumor-supporting macrophages.

## Introduction

Cancer cells are confronted with cells of the immune system throughout all phases of the disease, from early carcinogenesis to tumor progression and metastasis. In this respect, macrophages play a prominent role and have been shown to actively contribute to each cancer stage (1, 2). During chronic inflammations, driven for example by auto-immunity or persistent infections, macrophages play a central role as perpetuators of the disease, thereby producing cytotoxic and mutagenic compounds (for example, reactive oxygen and nitrogen species) that cause collateral damage to the surrounding tissue, leading to carcinogenesis. At metastatic sites, macrophages also cross-talk with the surrounding tissue, priming it for the arrival of circulating cancer cells and aiding those cancer cells to invade the metastatic niche (1). However, most evidence is available for a tumor-promoting role of macrophages within the primary tumor microenvironment.

The ecosystem of solid tumors is quite dynamic and complex, encompassing multiple non-transformed cells that are essential for the organoid behavior of a tumor. These cells include fibroblasts – responsible for the production of extracellular matrix, endothelial cells (EC) – responsible for blood vessel formation, and multiple hematopoietic cell types. The immune composition (also termed the immune contexture) of a tumor, primarily defined as the type, density, functional orientation, and location of adaptive immune cells, changes at each tumor stage and can independently predict disease outcome (3, 4). In particular, signatures associated with Th1 and cytotoxic T-cell responses predict a prolonged disease-free survival (3). In addition, tumors contain sizeable populations of myeloid cells, including neutrophils, eosinophils, dendritic cells, and especially macrophages. In extreme cases, tumor-associated macrophages (TAM) could represent up to 50% of the tumor mass. For long, this was considered to be an indication of anti-tumor immunity considering the inherent phagocytic and cytotoxic properties of macrophages. By now, it is clear that these protective properties of macrophages are suppressed in the tumor microenvironment and can only be reinvigorated upon therapeutic intervention. For example, macrophage-mediated cancer cell phagocytosis, resulting in tumor shrinkage, can be restored upon blockade of CD47, a “don’t eat me” signal overexpressed by most cancer cells (5). Along the same line, macrophage-dependent tumor cytotoxicity via nitric oxide can be stimulated by IL-2/anti-CD40 immunotherapy (6). However, when untreated, a significant link between TAM number/density and a poor prognosis becomes evident in most tumor types, illustrating the clinical significance of these cells (7, 8). This is mechanistically explained by the contribution of TAM to cancer cell invasion and metastasis, to angiogenesis, and to immunosuppression [reviewed in Ref. (1, 9)]. Moreover, TAM contribute to tumor relapse following tumor irradiation, the administration of anti-angiogenic, and vascular-disrupting agents and some forms of chemotherapy (10, 11). Thus, it becomes clear that dynamic changes in the phenotypes of macrophages occur during tumor initiation, progression, and metastasis and that subpopulations of TAM are responsible for distinct tumor-promoting activities (1, 2, 12). However, several open questions remain on the mechanisms behind the generation of TAM subsets that are crucial for defining therapeutic approaches in cancer: which is the network of signaling molecules, transcription factors, epigenetic mechanisms, and posttranscriptional regulators that underlie the different activation states? Do these activation switches involve recruitment of circulating precursors or the re-education of cells *in situ*? How reversible is TAM polarization?

In this review, we discuss several of these mechanisms and open questions, and we draw parallelisms with macrophages associated with other tissues in pathological and non-pathological conditions.

## TAM Diversity in Terms of Ontogeny

Peripheral blood monocytes were long thought to be obligatory intermediates in the differentiation of tissue macrophages. However, recent evidence demonstrated that several, if not most, organ-resident macrophages originate early in life, either during embryogenesis or shortly after birth. Indeed, fate mapping analyses revealed that liver Kupffer cells (KC), lung alveolar macrophages, peritoneal macrophages, epidermal Langerhans cells, and brain microglia were derived from primitive precursors and were self-maintained locally under steady state without a significant input from circulating monocytes (13–17). The exact nature of this primitive precursor is still a matter of some debate and includes fetal liver monocytes (15, 17) and yolk sac-derived macrophages (18). Obviously, cancer is a deviation from the steady state and it is therefore unclear to what extent *bona fide* tissue-resident macrophages contribute to tumor development and progression. Solving this issue is not trivial considering the paucity of markers to discriminate between tissue-resident and tissue-recruited macrophages.

### Evidence for the involvement of tissue-resident macrophages in cancer

In this section, we will focus on the role of KC and microglia during hepatocellular carcinoma (HCC) and glioma development and progression, respectively. These two cases illustrate the complexity of analyzing macrophage ontogeny during cancer and could be exemplary for tumors in other organs.

During diethylnitrosamine (DEN)-induced hepatocarcinogenesis, KCs were reported to stimulate tumorigenesis through the activation of pro-inflammatory receptors such as TREM-1 (19) and the secretion of hepatomitogens such as TNF and IL-6 (20). As a matter of fact, in this model, the gender differences in tumor incidence are dependent on the estrogen-regulated difference in IL-6 production by male versus female KCs (21). Though originally thought of as a non-inflammatory regimen, it is now clear that DEN treatment provokes local and systemic inflammatory responses characterized by the intrahepatic induction of distinct inflammatory chemokines and influx of macrophages and T cells (22). It remains therefore unclear whether DEN-mediated hepatocellular carcinogenesis truly relies on tissue-resident KCs or rather on liver-recruited macrophages. In established HCC tumors from patients, CD68^+^ monocytes/macrophages were amongst the most abundant inflammatory cells, comprising two main subpopulations: small HLA-DR^high^ IL-10^low^ cells in peritumoral stroma, reminiscent of newly recruited monocytes, and larger HLA-DR^low^ IL-10^high^ cells in cancer nests resembling mature macrophages (23). These populations were suggested to promote tumor progression by fostering immune privilege through the expression of PD-L1 (24, 25) and the induction of regulatory T cells (Treg) (26). However, whether any of these populations comprise KCs is questionable. Indeed, when applying rather strict parameters for the identification of these cells (CD68^+^, present in the blood space of cancer tissue, stellate or spindle shape), it turns out that KCs are underrepresented in cancerous tissue compared to adjacent healthy tissue and their numbers steadily decrease in later tumor stages (27). Finally, evidence exists for the implication of KCs in liver metastasis formation by colorectal cancer (CRC) cells (28). When CRC cells escape the primary tumor, they typically end up in the portal vein and the liver sinusoids, where they are confronted with KCs. On the one hand, KCs might present tumoricidal activity thereby limiting metastasis. However, when cancer cells are not immediately killed (for example, if too many of them arrive in the sinusoids and KCs become saturated) KCs might actually promote metastasis by trapping CRC cells and activating the endothelium for extravasation.

In the brain, microglia can mitigate the tumor-forming capacity of brain tumor initiating cells (BTIC) that contribute to the genesis or recurrence of gliomas, but that capacity is lost when tumors become established (29). At that later stage, microglia are able to stimulate glioblastoma cell invasion via epidermal growth factor (EGF) secretion (30). Similarly, during glioma progression, TAM contribute to tumor expansion via MT1-MMP (31), but the relative contribution of microglia versus bone marrow-recruited macrophages is unclear. Both populations are indeed present in gliomas, rather vaguely described as CD11b^+^CD45^low^ (for microglia) and CD11b^+^CD45^high^ (for recruited macrophages) illustrating that these cells are difficult to discern (32). In fact, a surface marker was identified – F11R – that typifies glioma-associated macrophages irrespective of their ontogeny (33), suggesting that the tumor microenvironment might overrule ontogenic differences.

In conclusion, both tissue-resident and -recruited macrophages might coexist in tumors, but their respective contribution to various aspects of tumor progression and dissemination remains to be established and awaits better tools to discern ontogenically distinct macrophage populations.

### Evidence for the involvement of tissue-recruited macrophages in cancer

As mentioned in the previous section, bone marrow-derived progenitors can contribute to the TAM population. This is expected to be especially prominent under (chronic) inflammatory conditions and injury, during which the tissue macrophage pool is typically reinforced by the influx of monocytes, as demonstrated in liver (34), skin (35, 36), brain (37), colon (38), and other tissues. Peripheral blood monocytes consist of two main populations, known as inflammatory or classical monocytes (Ly6C^high^ CX_3_CR1^low^ CCR2^high^ in mouse and CD14^++^CD16^−^ in man) and patrolling or non-classical monocytes (Ly6C^low^ CX_3_CR1^high^ CCR2^low^ and CD14^+^CD16^++^), whereby the patrolling population can be derived from inflammatory monocytes (38). Current evidence suggests that the inflammatory/classical monocytes are rapidly recruited into inflamed tissues, while patrolling/non-classical monocytes rather survey the intravascular vessel wall and mediate the elimination of stressed or damaged EC (39). However, in some pathologies, patrolling monocytes do make it into the tissues, where they rather contribute to a healing response (40).

Tumors have been described as “wounds that do not heal” (41) and are often linked with chronic inflammation, resulting in a prominent monocyte infiltration in the tumor microenvironment. However, the monocyte subset that is recruited to the primary tumor and the involvement of the CCL2-CCR2 chemokine axis in this phenomenon appears to be model- and tumor type-dependent. In the case of transplantable mammary and lung carcinomas, a CCR2-driven recruitment of Ly6C^high^ monocytes gives rise to different TAM subpopulations (42–45) Similar findings were obtained in the K14-HPV/E_2_ transgenic model of cervical carcinogenesis (46) and a Kras^LSL/G12D/+^; p53^fl/fl^ conditional genetic mouse model of lung adenocarcinoma (47). Moreover, inflammatory monocyte recruitment to human pancreatic tumors decreases patient survival, in agreement with the fact that patients with tumors that exhibit high CCL2 production have a worse prognosis (48). CCL2-mediated monocyte recruitment to the tumor is also important in patients suffering from follicular lymphoma (49). Overall, a high CCL2 expression level is correlated with a worse outcome in many cancer types, suggesting that CCR2^+^ inflammatory monocytes often function as tumor-promoting cells. An interesting issue is the origin of these tumor-infiltrating monocytes. The bone marrow is classically seen as the major source of peripheral blood monocytes, whereby CCR2 is critical for Ly6C^high^ monocyte emigration to the blood (50). Recently, however, the spleen was discovered as a reservoir for monocytes that can be mobilized in case of emergency. During tumor growth, angiotensin II overproduction drives hematopoietic stem cell retention in the spleen and the local differentiation of monocytes (51). Subsequently, splenic Ly6C^high^ monocytes are recruited to the tumor in a CCL2/CCR2-dependent fashion, where they contribute significantly to the TAM pool and tumor progression (47). In the MMTV-PyMT transgenic model of mammary adenocarcinoma development, Ly6C^high^ monocytes are only recruited to lung metastases where they promote extravasation of cancer cells, whereas primary tumors are infiltrated with Ly6C^low^ monocytes (52). Alternatively, a sequential infiltration of both monocyte subsets during distinct tumor stages is also possible, as shown in the ID8 ovarian carcinoma model (53). Finally, it should be noted that monocytic myeloid-derived suppressor cells (MO-MDSCs) strongly resemble Ly6C^high^ monocytes (54). Within the tumor, MO-MDSCs rapidly differentiate into macrophages when exposed to hypoxia, in a HIF-1α-dependent way (55).

### Evidence for the involvement of intra-tumoral TAM proliferation

Self-maintenance of tissue-resident macrophages is the consequence of low-grade proliferation. The proliferation rate of these cells can be enhanced by CSF-1/M-CSF and/or IL-4 under conditions of helminth infection (56, 57), a healing response (58), atherosclerosis and possibly other types of inflammation. Remarkably, relatively little information is available on the proliferative capacity of TAM. In the transplantable Lewis lung carcinoma and TS/A breast carcinoma models, cell cycle analysis did not reveal significant levels of TAM proliferation (42, 44). In the former model, parabiotic experiments demonstrated that the tumor microenvironment may support monocyte/macrophage survival, rather than proliferation (45). However, one study in human breast cancer patients established the presence of PCNA^+^CD68^+^ cells in tumors, suggestive of proliferating TAM (59). Proliferating TAM were significantly correlated with high grade, hormone receptor negative tumors, and a basal-like subtype, and were predictors of recurrence and survival. It is therefore conceivable that TAM proliferation becomes more prominent if tumor inflammation and the rate of monocyte influx are less prominent.

## TAM Diversity in Terms of Activation State

Macrophages demonstrate a high degree of plasticity in response to local cues from the microenvironment and can assume a spectrum of roles required for tissue homeostasis, ranging from host defense against infectious agents, to tissue development, wound healing, and immune system regulation (60). Macrophage activation was originally categorized on a linear scale, where the two extremes were the classically activated pro-inflammatory M1 macrophages, induced by IFNγ and toll-like receptor (TLR) ligands (M1), and the alternatively activated anti-inflammatory macrophages, induced by IL-4/IL-13, mirroring and mediating the polarized Th1-Th2 responses, respectively [reviewed in Ref. (60–62)]. The M1/M2-classification, although conceptually useful, tends to oversimplify the functional diversity of macrophages (63, 64). It has been proposed that macrophages can also undergo innate activation (TLR ligands without IFNγ), leading to pro-inflammatory but poor antigen presenting cells, or turn into regulatory cells (TLR ligands with anti-inflammatory mediators such as prostaglandins or immune complexes) that are IL-10^high^IL-12^low^ but good antigen presenters (65, 66). Along the same line, an alternative macrophage classification has been proposed, based on the three fundamental macrophage functions that are involved in maintaining homeostasis: host defense, wound healing, and immune regulation. These three basic types of macrophages would be able to blend into many other “shades” of activation that remain to be identified (64).

### Evidence for differentially activated TAM within tumors

Clearly, cells of the monocyte/macrophage lineage are characterized by their high diversity and plasticity. According to a model of functional adaptivity proposed by Stout et al., macrophages are capable not only of adapting to microenvironmental signals by mounting different functional patterns, but also of changing their functional phenotype in response to progressive variation of these signals (67). In cancer, too, it is becoming clear that macrophages are heterogeneous and, depending on the tissue and type of tumor, the stage of tumor progression and location within the tumor tissue, different subpopulations of macrophages may differ considerably in terms of function and M1/M2 phenotype (2, 68–70). Firstly, macrophages in their capacity of prominent inflammatory M1 cells play a non-redundant role during chronic inflammation-associated carcinogenesis. For example, blocking the CCL2-mediated recruitment of macrophages to the chronically inflamed colon resulted in a reduction of colitis-associated carcinogenesis (71). Along the same line, CCL2 upregulation by prostaglandin E_2_ during *Helicobacter pylori* infection is needed to recruit macrophages to the stomach and initiate gastric tumor formation (72). Interestingly, during skin carcinogenesis, fibroblasts were shown to be obligatory for maintaining the CCL2-mediated recruitment of macrophages (73). Inflammatory and anti-inflammatory signaling pathways determine the carcinogenic potency of macrophages. Indeed, a myeloid cell-specific deficiency in NF-κB activity reduces tumor formation (74), while a myeloid cell-specific deficiency of STAT3 results in the spontaneous development of colitis triggered by the gut microflora and leads to an enhanced rate of tumor formation in inflamed regions (75).

Also in established tumors, macrophages may promote the progression of the disease. This is exemplified by the fact that TAM presence is correlated with poor prognosis in many types of cancers, such as Hodgkin lymphoma, glioma, cholangiocarcinoma, and breast carcinoma (7, 76–79) while some exceptions like colon cancer (80, 81) have been reported. In fact, more recent research has suggested that the phenotype and activation state of TAMs, rather than their absolute number, is more informative for patient prognosis (82). For instance, infiltration of CD40^+^ macrophages in CRC and of CD14^+^CD163^−^ macrophages in cervical cancer were associated with a favorable prognosis (81, 83). As stated earlier, it is still not fully resolved whether TAM diversity results from the maturation of unique monocytic precursors or from differences in micro-anatomical factors (42). However, it is highly likely that the different activation states of TAM subsets reflect responses to dynamic local microenvironmental cues within the tumor.

**Figure 1:**
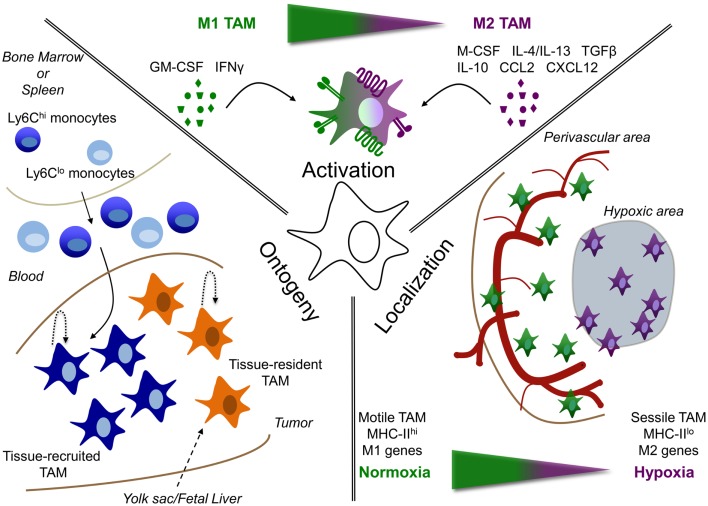
**Tumor-associated macrophage heterogeneity depends on ontogeny, activation, and localization**. TAM are heterogeneous depending on their: (i) ontogeny, (ii) activation, or (iii) localization. (i) TAM could either be derived from the self-renewing tissue-resident macrophage pool (tissue-resident TAM), whose precursors are formed early in life in the yolk sac or fetal liver; or can be derived from circulating monocytes, termed tissue-recruited TAM. In addition, maintenance or accumulation of TAM could be due to proliferation in the tumor. (ii) Depending on the signals, they receive from their microenvironment, TAM can be either more M1 or M2 activated or display a mixed activation state. (iii) In addition, the localization of TAM can play a role in their phenotype. For example, molecular and functional differences are observed between TAM residing in normoxic perivascular areas or hypoxic areas.

The activation state of macrophages in cancer depends on the stage of tumor development. Indeed, at least in some models of carcinogenesis in the mouse, tumor progression is associated with a phenotypic switch from M1 to M2 macrophages (84). This has led to the consensual view that, in sites of chronic unresolved inflammation, macrophages initially triggered by a pathogen or tissue stress, recruit monocytes that develop into additional inflammatory M1-like macrophages. While this may initiate the first steps of carcinogenesis, as discussed in the previous chapter, the inflammatory cascade may also contribute to the T-cell-mediated elimination and equilibrium phases during tumor progression (85). At later stages of tumor progression, recruited monocytes differentiate into macrophage subpopulations with an overall more M2-like, wound healing or “trophic” phenotype (low IL-12 expression, high IL-10 expression, and low tumoricidal activity) that promotes tissue remodeling and angiogenesis (1, 86). However, it should be realized that within these more M2-oriented cells, subpopulations exist with a somewhat different activation profile (42–44). In any case, a dynamic activation switch occurs among TAMs as the tumor develops, which may help explain the “mixed” activation state of TAM populations found in different established murine and human tumors (87–89). Alternatively, macrophages within established tumors may be exposed to opposing signals that underlie the different TAM activation states and subpopulations found within the same tumor (Figure 1).

Various pathways orchestrate the function of myelomonocytic cells, and are induced by signals from the tumor itself and from the stroma (such as fibroblasts and infiltrating leukocytes). These signals include:

### Myelopoietic growth factors

M-CSF (CSF-1) instructs the myeloid fate in hematopoietic stem cells and remains instrumental for the generation of monocytes and macrophages (90). Notably, M-CSF-driven macrophage differentiation leads to the expression of a substantial part of the M2 transcriptome, including expression of mannose receptor 1 (*Mrc1* or MMR) and scavenger receptor A (SR-A), while GM-CSF (CSF-2) rather induces an M1-type of activation (91–93).

A M-CSF response signature is often observed in primary tumors and corresponding metastases, correlating with tumor grade and worse prognosis (94–96). Accordingly, recent studies have reported high expression of M-CSF in peritumoral liver tissue, which was associated with macrophage density, intrahepatic metastasis, and poor survival after hepatectomy (97, 98). M-CSF, together with other tumor-derived factors such as CCL2, induces monocyte infiltration in tumors and macrophage activation toward a trophic pro-angiogenic phenotype (leading to what is called the angiogenic switch). Thus, genetic depletion of M-CSF in a spontaneous model of breast cancer formation (MMTV-PyMT) dramatically decreased macrophage infiltration and affected progression from non-malignant adenoma to malignant carcinoma (82). Accordingly, growth of transplantable tumors in M-CSF-deficient mice (*Csf1^op^/Csf1^op^*) is markedly impaired (99).

These findings have been therapeutically exploited, showing that M-CSFR inhibition via small molecule inhibitors or blocking antibodies decreases the presence of TAM in transplantable or spontaneous tumor models and leads to a reduced tumor angiogenesis and lymphangiogenesis (100–102). Hence, M-CSFR blockade prevents resistance to anti-angiogenic and radiation therapy (100, 103). Also in a mouse glioblastoma model, an inhibitor of M-CSFR significantly increased survival and regressed established tumor, which was in this case not due to TAM depletion, but rather to a decreased expression of M2 markers and an unopposed production of anti-tumoral GM-CSF and IFNγ (104). These data are in agreement with the notion that M-CSF can contribute to angiogenesis *in vivo* by inducing VEGF in macrophages through the MAPK/Erk pathway (105), while GM-CSF appears to exert opposite functions by re-educating macrophages to become anti-angiogenic through the secretion of soluble VEGFR1 (106). Hence, the intra-tumoral M-CSF/GM-CSF balance could determine the M1/M2 TAM balance and consequently, the TAM effect on tumor growth. Finally, it should be noted that M-CSF is not only capable of differentiating macrophages with pro-tumor properties, but it also mediates the expression of activating Fc receptors, resulting in a tumoricidal function in the presence of tumor-targeting mAbs (107).

### Cytokines

#### The IL-4/IL-13/Stat6 pathway

IL-4 and IL-13 were shown to be crucial mediators in the stimulation of invasion or immunosuppression by TAM in several tumor types. Thus, IL-4, mainly produced by intra-tumoral CD4^+^ Th2 cells, modulates the TAM phenotype and induces them to secrete EGF, leading to EGF-R-dependent invasion and metastasis (108), as well as cathepsins B and S, shown to be critical for promoting tumor growth, angiogenesis, and invasiveness in several tumor models (109). Similarly, deficiency of IL-13, mainly produced by tumor-infiltrating NKT cells, was shown to result in a dominance of M1-type TAMs and resistance to metastasis (110). Both cytokines are recognized by receptors that share the IL-4Rα chain and activate the transcription factor Stat6, which in turn activates M2-type genes such as arginase 1 (*Arg1*) and *Mrc1* (111, 112). Accordingly, Stat6-deficiency in the hematopoietic compartment results in increased tumor immunosurveillance (113). IL-4 also induces c-Myc activity in human macrophages, which controls genes of M2 activation (*Scarb1, Alox15*, and *Mrc1*) (114), and c-Myc regulates the differentiation and the pro-tumoral activity of TAM (115). Furthermore, it suppresses transcription of M1-associated genes by inhibiting Stat-1 signaling via Socs1 upregulation (116) and by inducing epigenetic changes through upregulation of the histone demethylase JMJD3 (117).

Downstream of the Stat/Socs pathway, there is a panel of transcription factors, also induced via IL-4/Stat6, that orchestrate polarized activation. For instance, Stat6 synergizes with PPARγ (118) to induce oxidative metabolism genes, and with KLF4 to induce M2 genes such as *Arg1* and inhibit M1 genes such as TNF (*Tnf*) and iNOS (*Nos2*) via the sequestration of NF-κb coactivators (119). Considering that several PPAR agonists are already in clinical use for metabolic disorders, the precise contribution of PPARγ in TAM function and tumor development requires further investigation (120).

#### The IL-10/TGFb/Stat3 pathway

The anti-inflammatory cytokines TGFβ and IL-10 are produced by neoplastic cells, tumor fibroblasts, and tumor-infiltrating Treg and both cytokines activate the Stat3 pathway, leading to the expression of genes associated to an M2 phenotype, such as *IL-10, Tgfb1, Mrc1* (65, 112, 121). Accordingly, systemic administration of an anti-IL-10R antibody, in combination with local treatment with CpG, was shown to induce a shift in resident and recruited TAMs from the M2 into the M1-type, accompanied by tumor shrinkage (122). Similarly, inhibition of TGFβ signaling in combination with TLR7 ligation reprogramed the phenotype of TAMs toward an M1 tumoricidal phenotype, and this effect was accompanied by enhanced NF-κb nuclear translocation (123). In agreement with these results, TGFβ signaling in TAMs induces expression of high levels of the inactive serine/threonine kinase IRAK-M, which acts as a potent negative regulator of TLR signaling (124). Conversely, Stat3 can be inhibited by Socs3, as a result of IFN and TLR signaling and Notch activation (125).

Constitutive activation of the Stat3 signaling pathway has been observed in tumor cells as well as infiltrating cells, including TAMs (126). Importantly, production of angiogenic factors such as VEGF and bFGF by TAMs are induced by Stat3, and experiments with mice deficient for Stat3 in the myeloid compartment, showed that it is a crucial mediator for myeloid cell-induced tumor angiogenesis (127).

#### The Stat3/NF-κb interplay

An interesting interplay exists between Stat3 and NF-κb, both transcription factors being persistently activated in cancer and regulating a great number of genes important for cancer-promoting inflammation [reviewed in Ref. (128)]. Stat3 has been shown to maintain constitutive NF-κb activation in tumor-associated myeloid cells (129), and several inflammatory factors encoded by NF-κb target genes, notably IL-6 released by TAMs, are important Stat3 activators. Accordingly, a study with human cervical carcinoma lines showed that tumor-derived IL-6 and PGE_2_ were responsible for skewing monocyte differentiation toward a CD14^+^CD163^+^ M2-type phenotype, and that this was reversible upon co-incubation with Th1 cells (130). While Stat3 activation restrains anti-tumor immune responses by antagonizing Stat1-mediated expression of anti-tumor cytokines such as IFNγ and IL-1 (131, 132), NF-κb activation plays a dual role: it is crucial for inducing oncogenic inflammatory conditions, but also for generating anti-tumor responses [reviewed in Ref. (133)]. Thus, it has been proposed that while classical NF-κb activation plays a pro-inflammatory role in macrophages during early stages of tumor growth, in established tumors signals such as lymphotoxin, BAFF or CD40, can lead to alternative NF-κb activation (134). Furthermore, NF-κb p50 homodimers have been shown to play a key role in the orchestration of M2 responses (135). However, the role of classical NF-κb activation in TAMs remains controversial since inhibition of IKKβ (and therefore of NF-κB) was shown to promote an M1-like phenotype, whereas functional IKKβ/NF-κB activation maintained these cells in an alternative, tumor-promoting phenotype (88).

TNF, a cytokine that classically induces NF-κb activation, plays a role in promoting tumor progression by inducing monocytes to produce M-CSF (136) and by stimulating, together with IFNγ, expression of the negative costimulatory molecule PDL-1 (B7-H1) on macrophages (137), leading to suppressed cytotoxic T-cell responses (138). Additionally, in mouse and human colon cancer, TNF mediates upregulation of COX-2 by macrophages, greatly promoting carcinogenesis via PGE2 production (139, 140).

### Chemokines

#### CCL2

Expressed by macrophages, fibroblasts, endothelial, and tumor cells, CCL2 is one of the most frequently observed chemokines in a wide range of tumors and one of the main determinants of monocyte/macrophage recruitment. In addition, CCL2 has been shown to induce, together with IL-6, upregulation of *Mrc1* in CD11b^+^ human mononuclear cells, suggesting a polarization to an M2-type phenotype (141). However, the biological effect of CCL2 may be biphasic and more related to recruitment than polarization as suggested by a study where low-level CCL2 secretion by non-tumorigenic melanoma cells led to modest monocyte infiltration and stimulation of tumor formation due to increased angiogenesis, while high CCL2 levels were associated with massive monocyte infiltration into the tumor mass, leading to its destruction (142).

#### CCL5

CCL5 is not only produced by naive T cells but also by breast tumor cells, contributing to monocyte migration into tumor sites (143). Interestingly, CCL5 stimulates human monocytes to express CCL2, CCL3, CCL4, and CXCL8, all of them chemoattractants for myeloid cells (144). It also stimulates the expression of the receptor CCR1 on monocytes, which is recognized by numerous chemokines. Hence, activation of monocytes by chemokines leads to further recruitment of more monocytes into the tumor mass, as well as that of other leukocyte populations (145). Indeed, monocyte to macrophage differentiation involves CCR2 downregulation and increased expression of CCR1 and CCR5 (146, 147), suggesting a multistep navigation process whereby the initial CCR2-dependent recruitment of monocytes is followed by a CCR1/CCR5-dependent positioning within the tumor (148). This scenario is supported by the observation that, in hypoxic conditions, monocytes migrate poorly in response to CCL2 (149).

Other chemokines, including CCL3, CCL4, CCL8, and CCL22 (macrophage-derived chemokine), have been detected in ovarian tumors (150) and high levels of CXCL8 and CCL18 have also been found in ascitic fluids from patients with ovarian carcinoma (151). While the presence of these chemokines in the tumor mass has been correlated with the presence of macrophages, it still remains to be determined whether they play a role in the recruitment or in the maintenance of the TAM population in the neoplastic tissues.

### The CXCR4/CXCR7/CXCL12 axis

CXCR4 is one of the most ubiquitously expressed chemokine receptors and it is overexpressed in many human cancers, including breast cancer, ovarian cancer, melanoma, and prostate cancer [reviewed in Ref. (152)]. CXCR7, the other receptor for CXCL12 (SDF-1), is expressed by many tumor cell lines (153), and its role as scavenger or co-receptor for CXCL12 remains in debate. Although not expressed in normal blood leukocytes, CXCR7 expression was shown to be upregulated upon monocyte to macrophage differentiation, with a higher expression in M1 than in M2 phenotype, and to play a role in phagocytic activity (154). However, its role in TAM differentiation requires investigation. In any case, CXCL12 can modulate monocyte-macrophage differentiation toward a pro-angiogenic phenotype by upregulating VEGF and CCL1 and down-regulating RUNX3 (155). Furthermore, mixed M1/M2 macrophages described in colon cancer metastasis in liver promote cancer growth via a GM-CSF/HB-EGF paracrine loop that is enhanced by CXCL12 (156). Importantly, hypoxia drives expression of CXCL12 by EC (157) as well as of its CXCR4 receptor (158). Thus, in hypoxic regions of expanding tumors, CXCR4 receptor levels might be increased not only to facilitate TAM-mediated angiogenesis, but also tumor cell survival, local invasion, and escape from the primary tumor mass (159).

Finally, it is important to keep in mind that TAM polarization is not only affected by, but also significantly affects tumor-specific T-cell responses [reviewed in Ref. (60)]. For example, M1-type TAM produce cytokines (IL-6, IL-23, IL-1β, TNFα) that promote Th17 differentiation and maintain the chronic inflammatory environment (160). M2-like TAMs impair T-cell activation and effector functions via the secretion of immune-suppressive factors such as IL-10, PGE_2_, and arginase and the expression of inhibitory ligands such as PD-L1 (B7-H1). Furthermore, they enhance recruitment of Treg via production of CCL22 (161) and can drive differentiation of Treg via TGFβ (162). TAM that originate from MO-MDSC can also dampen T-cell responses by inducing CD4^+^ T-cell apoptosis in a Stat-1 dependent manner (163). In addition, it has been proposed that production of CCL20 by TAMs in hypoxic regions may exert a “sink”-like effect by attracting NKT cells where their viability and function are impaired (164).

## TAM Diversity in Terms of Intra-Tumoral Localization

Tumors are complex, developing organoid structures that contain several different ecosystems, such as cancer nests, peritumoral stroma, perivascular regions, and hypoxic regions. Hence, given the plasticity of these cells, the macrophage phenotype differs within different areas of the same tumor and distinct functions for these TAM subpopulations have been predicted (165).

A study by Wyckoff et al. showed that macrophages are present in large numbers at the margins of mouse mammary tumors, while fewer of these cells are found in association with blood vessels deeper in the tumors (166). Importantly, these perivascular TAM produce EGF, which attracts M-CSF-producing cancer cells, resulting in a coordinated migration and intravasation of the cancer cells at sites of high macrophage density (166, 167). As a matter of fact, the interaction between cancer cells, macrophages, and EC (tumor microenvironment of metastasis or TMEM) is predictive of metastasis formation in breast cancer patients (168). The migratory nature of perivascular macrophages is confirmed in a study by Kedrin et al., while only limited TAM migration was observed in avascular regions (169). Along the same line, spinning disk confocal microscopy on mammary carcinomas identified motile and sessile TAM subpopulations in different compartments of the tumor (170). Comparing the gene expression profile of the migratory TAM with the more sessile cells (which have a higher phagocytic capacity) demonstrated that most of the consensus M2 markers (171) were expressed at a higher level in the latter (172). Accordingly, upon labeling of tumor areas proximal to perfused vessels with Hoechst 33342 and sorting of perivascular Hoechst^+^ versus vessel-distal Hoechst^−^ macrophages, the latter were shown to express higher levels of M2 markers (173). The presence of differentially activated macrophages within the same tumor was further corroborated by studies discriminating between MHC-II^high^ and MHC-II^low^ TAM in different transplantable and transgenic tumor models, which are more M1- and M2-like, respectively, and which reside in distinctively oxygenated tumor regions (42–44). MHC-II^low^ TAM are found in the most hypoxic tumor regions and express clearly higher levels of M2 markers such as CD124 (IL-4Rα), stabilin-1, CD204 (SR-A), and CD206 (MMR). As a matter of fact, molecular imaging of CD206^high^ TAM using anti-CD206 nanobodies has been proposed as a strategy to visualize hypoxic areas in tumors (43). Similarly, MHC-II^high^ and MHC-II^low^ TAM subsets are present in different regions of human HCC, whereby MHC-II^low^, but not MHC-II^high^, TAM score positive for IL-10, suggestive of a more M2-like orientation (23).

### The influence of hypoxia on TAM heterogeneity

Considering the presence of differentially activated macrophages in perfused and non-perfused tumor regions, hypoxia could be an important determinant of the TAM phenotype. Indeed, most solid tumors contain regions of chronic or cycling hypoxia (0.1–2% O_2_) resulting from an abnormal vascularization in combination with a high metabolic activity (174). Hypoxia is known to influence the behavior of multiple stromal cell types in tumors, including macrophages, but how oxygen tension shapes the inflammatory response of macrophages and modulates specific differentiation states is less well studied (175, 176).

A recent study demonstrated that the expression of the most prominent M2 markers – CD206, CD124, and *Arg1* – is not altered in TAM subsets from better oxygenated tumors grown in *Phd2*-haplodeficient mice, which display vessel normalization (44). Rather, reduced hypoxia downregulated the expression of several pro-tumoral genes, involved in glycolysis, angiogenesis, and metastasis, specifically and solely in the hypoxic MHC-II^low^ TAM subset (44). These data suggest that hypoxia is not the main driving force behind the typical M1-like/M2-like TAM activation profiles *per se*, but M2-like TAM preferentially home to hypoxic areas where the pro-tumoral activities of these cells are boosted. Formal proof for this concept came from the finding that neuropilin-1 (Nrp-1) expression in macrophages is crucial for the migration of these cells into hypoxic areas and for the induction of their pro-angiogenic and immunosuppressive activities (176). Semaphorin3A, but not VEGF, was demonstrated to be the main chemoattractant, signaling through a Nrp-1-dependent PlexinA1/PlexinA4/VEGFR1 receptor complex. A macrophage-specific Nrp-1 deficiency leads to an accumulation of TAM in normoxic regions, maintaining more M1-like features such as high NO secretion, cytotoxic activity, and T-cell stimulatory capacity (176). As a result, tumors grow significantly slower, highlighting the importance of a hypoxia-dependent programing of macrophages for tumor progression.

How the initial M1/M2 dichotomy of TAM subsets (independent from intra-tumoral oxygen levels) is regulated is still unclear. One possibility is an interaction between infiltrating monocytes and EC, whereby EC stimulate macrophage differentiation and an M2-like activation through M-CSF (177). Angiopoietin-2, produced by activated EC, upregulates its receptor Tie2 on macrophages and maintains these M2-like cells in the neighborhood of blood vessels, at least for a while (178). Blocking Ang-2 redistributes these macrophages. It is therefore conceivable that a temporary interaction with EC instructs the M2 phenotype (CD206^high^) and that these cells, upon release, move toward the hypoxic areas where additional M2-like features (such as angiogenic activity and immunosuppressive activity) are strengthened.

The gene regulation of pro-tumoral factors under hypoxia is likely to be mediated by the transcription factors HIF-1α and HIF-2α, considering the implication of both in regulating hypoxic adaptation in macrophages (179). In the transgenic PyMT mammary tumor model, a myeloid cell-specific deficiency in HIF-1α results in a hampered Arg1 and iNOS expression in TAM linked with an abolished T-cell suppressive capacity (180). HIF-2α has also been shown to regulate Arg1 and to correlate with tumor microvessel density, and could therefore also contribute to the pro-tumoral profile of hypoxic TAM (181, 182).

Together, these data support the notion that a reprograming of TAM toward a more M1-like phenotype is likely to be beneficial, as monotherapy, but especially in combination with immunotherapy. Blocking Nrp-1 seems to be a logical approach, and monoclonal antibodies preventing Nrp-1 interaction with either Semaphorin3A or VEGF have indeed proven their anti-tumoral potential (183). Another strategy is to apply low-dose irradiation to the tumor, which normalizes the vasculature and induces iNOS^+^ M1-like TAM that orchestrate CTL recruitment into tumors by stimulating endothelial activation and Th1 chemokine production (184). Along the same line, low-dose anti-VEGFR2 therapy causes vessel normalization and perfusion along with an enhanced M1 polarization of TAM, allowing a better outcome in combination with a tumor vaccine (173). More direct ways to alter the TAM phenotype include anti-CD40 immunotherapy (6), the combination of the TLR9 ligand CpG with blocking anti-IL-10R antibody (122), treatment with encapsulated IL-12 (185), or with a redox-active copper chelate (186).

## TAM Heterogeneity: Parallels with Other Tissues

**Figure 2:**
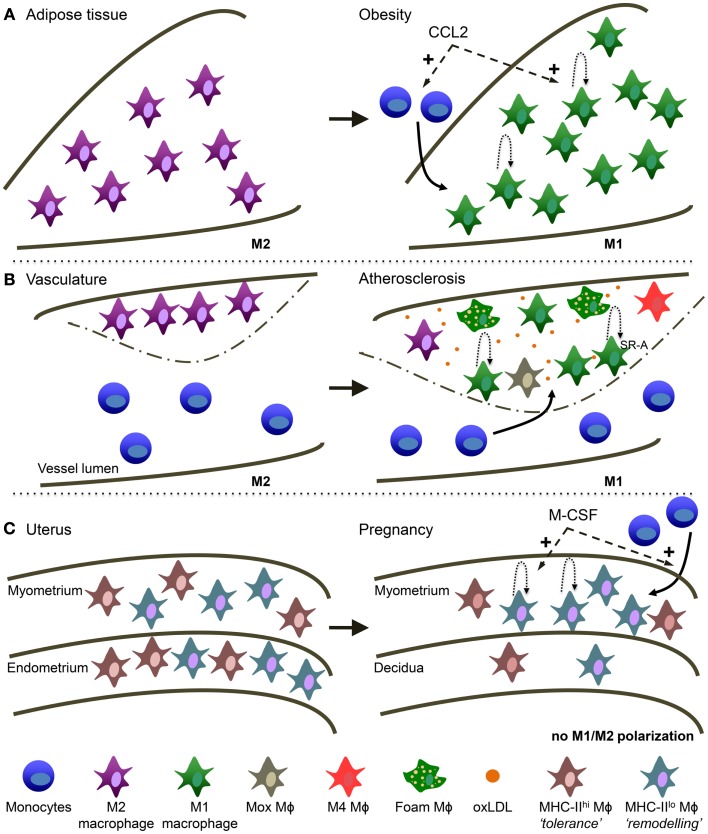
**Macrophage heterogeneity in different tissues is reminiscent of TAM heterogeneity**. **(A)** *Obese adipose tissue (AT)*. AT macrophages switch from a more M2 activation in healthy conditions to M1 activation in obese AT. During obesity, CCL2 is involved in monocyte recruitment to the AT and the stimulation of local macrophage proliferation. **(B)** *Atherosclerosis*. Macrophages switch from a more M2 activation in the healthy vessel wall to M1 in the atherosclerotic plaque. Macrophage accumulation in the lesion is due to monocyte recruitment and local macrophage proliferation mediated by SR-A. oxLDL is highly present and is a main trigger of macrophage activation in the lesion. In addition, several other types of macrophages have been described in atherosclerosis: “Mox,” “M4” and the typical foam macrophages. **(C)** *Maternal-fetal interface*. In the non-pregnant uterus MHC-II^low^ and MHC-II^high^ macrophages are evenly distributed over the myometrium and endometrium. In the pregnant uterus macrophage numbers decline in the endometrium (now called decidua) and MHC-II^low^ macrophages preferentially accumulate in the myometrium. M-CSF is involved in both the attraction of monocytes as well as macrophage proliferation.

Macrophage heterogeneity in response to different activation cues has been appreciated in other tissues and may underlie different pathological and non-pathological conditions (Figure 2).

### Obese adipose tissue

While in the lean state most of the macrophages present in the adipose tissue (AT) have an anti-inflammatory M2 phenotype, obesity induces an accumulation of a novel F4/80^+^CD11c^+^ population with increasing expression of M1-type genes such as TNF and iNOS (187), leading to chronic low-grade inflammation and insulin resistance [reviewed in Ref. (188)]. As with tumors, the contribution of recruited versus tissue-resident macrophages in obese AT requires further investigation. It has been proposed that AT macrophages (ATM) originate by recruitment of blood monocytes (189) and progressive polarization to M1 macrophages in response to tissue-derived signals (190). It has even been suggested there may be preferential homing of a subpopulation of monocytes to AT via recognition of the macrophage galactose C-type-lectin 1 (MGL1) (191). However, a very recent study in the ob/ob obese mouse model shows that a major fraction of ATM proliferates in a CCR2-dependent manner and contributes to AT inflammation independently of monocyte recruitment (192). Thus, the CCL2/CCR2 axis not only is the major chemokine/receptor pair involved in ATM recruitment [reviewed in Ref. (193)] but also in enhancing their proliferation. Accordingly, most studies show that deficiency or pharmacological inhibition of CCR2 decreases ATM and protects from insulin resistance (187, 194, 195). CCL2 is secreted by obesity-associated dysfunctional adipocytes, which also upregulate IL-6 and leptin, while down-regulating adiponectin [reviewed in Ref. (196)]. Interestingly, adiponectin, highly expressed by lean but not obese adipocytes, has recently been shown to be a key factor for M2 polarization via the Stat6 pathway (197). PPARγ, induced via the Stat6 pathway as discussed for TAM, seems to play a key role in ATM polarization, and short-term treatment with PPARγ activators promoted infiltration of M2-type macrophages into AT with an effect on AT morphology (198).

However, as with tumors, the M1/M2-like dichotomy is probably an oversimplification, and macrophages with a mixed M1/M2 profile have been found, particularly during the first stages of diet-induced obesity (199). Furthermore, while ATMs are also abundant in another metabolic disease, lypodystrophy, they have a distinct phenotype and do not appear to be involved in the pathogenesis of insulin resistance (200).

Interestingly, a recent study has shown that human ATMs display activation of cancer-related pathways (201). These data are particularly intriguing in the light of recent epidemiological studies that have shown a clear association between obesity and increased risk for a wide range of cancers (202). The relative contribution of endocrine, metabolic, and inflammatory mediators in the exacerbation of tumor growth and progression remain to be explored, as well as the influence of obesity-associated adipocyte dysfunction on the tumor microenvironment and TAM heterogeneity.

### Atherosclerotic lesions

Early studies performed in the apolipoprotein E (ApoE)-deficient mouse model of atherosclerosis suggested that macrophages promote atherosclerosis (203). In fact, at early stages of the disease, they favor lesion progression very likely through unregulated uptake of oxidized low-density lipoprotein (LDL) particles that activate innate immune receptors and trigger the expression of inflammatory cytokines, proteases, chemokines, and costimulatory molecules. However, at later stages they may counter lesion expansion via Mertk-mediated efferocytosis of apoptotic bodies (204).

Recently, in murine plaques, Arg1 and Arg-2 were described as M2 and M1 markers, respectively (205) and a transition of Arg1^+^ to Arg-2^+^ cells was described, suggesting an M2 to M1 switch during plaque progression. Interestingly, the balance between M-CSF to GM-CSF seems important during this process [reviewed in Ref. (206)]. Indeed, there is growing evidence that the balance of macrophages in the plaque is dynamic and that both macrophage numbers and their phenotype influence plaque fate (207). Therefore, as with tumors, macrophage heterogeneity in atherosclerosis is an accepted concept. However, as with tumors, the M1/M2 division is an oversimplification and besides traditional M1 and M2 macrophages, mixed M1/M2 as well as macrophages with a totally different phenotype have been observed. For instance, a macrophage population that shows a strong induction of the anti-oxidant response via upregulation of the transcription factor Nrf2 has been reported in advanced lesions and called “Mox” macrophages (208). CXCL4, present in artherosclerotic lesions can induce so-called “M4” macrophages, with low expression of scavenger receptors and increased levels of cholesterol efflux transporters (209). In addition, macrophages can also internalize oxidized LDLs via scavenger and oxLDL receptors, converting into foam cells, the hallmark of the atherosclerotic lesion. In fact, hypoxia in the lesions enhances lipid uptake by macrophages (210) via upregulation of the of the oxLDL receptor Lox-1 in a HIF-1α-dependent manner (211). Similarly, CXCL12 enhanced the phagocytic capacity of CXCR7-positive macrophages observed in lesions (154). Finally, as with ATM, a recent study showed considerable local macrophage proliferation that was dependent on expression of SR-A (212).

### Maternal-fetal interface

The mouse uterus contains two abundant populations of macrophages, defined by F4/80^+^ MHC-II^high^ and F4/80^+^ MHC-II^low^ surface phenotypes that are distributed evenly between the myometrium and the endometrium (213). Reminiscent of what has been described in TAM, MHC-II^low^ cells expressed higher levels of genes associated with an angiogenic, tissue remodeling, and repair phenotype, such as CD163, Stab1, and Mrc1. In contrast, the MHC-II^high^ cells expressed high levels of M1-type chemokines such as CCL5, CXC3CL1, and CCL17. During pregnancy, high levels of M-CSF expression in the myometrium were shown to induce macrophage proliferation and stimulate extravasation of Ly6C^high^ monocyte precursors in a CCR2-dependent manner (213). Importantly, M-CSF activity in the myometrium inhibited macrophage maturation and thus, promoted the accumulation of MHC-II^low^ cells. In a remarkably comparable way, decidual macrophages in humans comprise around 20% of leukocytes and also divide in two subsets: CD14^+^ CD11c^high^ and CD14^+^ CD11c^low^ macrophages (214). Similar to their MHC-II^high^ murine counterparts, decidual CD11c^high^ human macrophages were shown to express genes associated with lipid metabolism and inflammation. Moreover, these cells were more efficient in antigen processing and presentation as well as IL-10 secretion, suggesting they may contribute to tolerance induction toward fetal antigens via CD1-mediated presentation of lipids (214). On the other hand, the CD11c^low^ cells were positive for CD209 (DC-SIGN) and CD304 (Nrp-1) (215) and expressed genes associated with extracellular matrix formation and tissue growth, suggesting a role in maintenance, and growth of uterine muscle cells (214). However, both populations secreted both pro- and anti-inflammatory cytokines (214) and thus do not fit in the conventional M1/M2-classification. Finally, decidual macrophages likely also contribute to parturition involving the activation of inflammatory pathways, although the population(s) involved remain poorly defined and it is not known whether the constitutive IL-10 production observed during the first trimester is reduced in late gestation [reviewed in Ref. (216)].

Thus, in parallel with what is observed in tumors, the maternal-fetal interface is associated with an immune-suppressive, wound-healing microenvironment that guarantees the survival of the semi-allogeneic fetus, while ensuring uterine spiral artery remodeling and placental growth. However, as with tumors, there are different macrophage phenotypes in the uterus that exert different functions, and their numbers as well as their activation/polarization status are influenced by environmental cues – in particular M-CSF activity levels – that vary over time and according to the location.

## Conclusion

This review underlines the diversity of macrophage phenotypes and functions in tumors, which is very likely a result of their great plasticity in response to changing environmental cues as the tumor progresses. Furthermore, different micro-anatomical sites within the tumor may attract and instruct different macrophage subpopulations. As a result, macrophages may be exposed to different and sometimes opposing signals that underlie the different macrophage activation states and subpopulations found within the tumor. The importance of certain TAM subsets and their associated molecular armamentarium for tumor progression should not be underestimated. Indeed, it remains a remarkable observation that blocking the function of one molecule (e.g., Nrp-1) in one cell type (the macrophage) is able to dramatically impact the behavior of a very complex tissue such as a tumor (176). This suggests that macrophages are indeed central players in tumor biology and that the development of therapeutic strategies to interfere with their functions is of major relevance. Directing such therapies against the most tumor-promoting macrophage populations, while leaving anti-tumoral macrophages unharmed, seems to be the most promising way forward. In this respect, converting tumor-promoting M2-like TAM into anti-tumoral M1-like TAM can be achieved via several strategies: (1) preventing macrophages from entering hypoxic areas via Nrp-1 blockade enhances their M1 profile and reduces pro-tumoral activities (176). Notably, anti-Nrp-1 antibodies are currently in clinical trial for cancer therapy (217); (2) inhibiting M2-stimulating triggers via blocking antibodies or small molecule inhibitors. A non-limiting series of examples include the use of anti-M-CSFR antibodies or inhibitors (112), anti-IL-10R (130), COX-2 inhibitors (218), GTP cyclohydrolase inhibitors (219), Reg3β blockade (220), and others; (3) promoting M1-stimulation via triggering of TLR3 signaling (221), the administration of IL-12 (185), histidine-rich glycoprotein (222), Sorafenib (223), and other compounds.

Interestingly, the co-existence of distinct macrophage phenotypes within the same tissue is also seen in non-cancerous conditions, both in pathological and non-pathological settings and even under steady state. Hence, recent transcriptomic efforts showing a high diversity between macrophages from distinct tissues (224) could be extended to macrophage subsets within the same tissue and is likely to refine the concept of tissue-resident macrophages.

## Conflict of Interest Statement

The authors declare that the research was conducted in the absence of any commercial or financial relationships that could be construed as a potential conflict of interest.
